# Modeling Wheezing Spells Identifies Phenotypes with Different Outcomes and Genetic Associates

**DOI:** 10.1164/rccm.202108-1821OC

**Published:** 2022-01-20

**Authors:** Sadia Haider, Raquel Granell, John Curtin, Sara Fontanella, Alex Cucco, Stephen Turner, Angela Simpson, Graham Roberts, Clare S. Murray, John W. Holloway, Graham Devereux, Paul Cullinan, Syed Hasan Arshad, Adnan Custovic

**Affiliations:** ^1^ National Heart and Lung Institute, Imperial College London, London, United Kingdom;; ^2^ Medical Research Council Integrative Epidemiology Unit, Population Health Sciences, Bristol Medical School, University of Bristol, Bristol, United Kingdom;; ^3^ Division of Infection, Immunity and Respiratory Medicine, School of Biological Sciences, Faculty of Biology, Medicine and Health, University of Manchester, Manchester Academic Health Science Centre, Manchester, United Kingdom;; ^4^ Royal Aberdeen Children’s Hospital National Health Service Grampian, Aberdeen, United Kingdom;; ^5^ Child Health, University of Aberdeen, Aberdeen, United Kingdom;; ^6^ Human Development and Health and; ^10^ Clinical and Experimental Sciences, Faculty of Medicine, University of Southampton, Southampton, United Kingdom;; ^7^ National Institute for Health Research Southampton Biomedical Research Centre, University Hospitals Southampton National Health Service Foundation Trust, Southampton, United Kingdom;; ^8^ David Hide Asthma and Allergy Research Centre, Isle of Wight, United Kingdom; and; ^9^ Clinical Sciences, Liverpool School of Tropical Medicine, Liverpool, United Kingdom

**Keywords:** wheezing phenotypes, asthma, latent class, genetics, 17q12–21

## Abstract

**Rationale:**

Longitudinal modeling of current wheezing identified similar phenotypes, but their characteristics often differ between studies.

**Objectives:**

We propose that a more comprehensive description of wheeze may better describe trajectories than binary information on the presence/absence of wheezing.

**Methods:**

We derived six multidimensional variables of wheezing spells from birth to adolescence (including duration, temporal sequencing, and the extent of persistence/recurrence). We applied partition-around-medoids clustering on these variables to derive phenotypes in five birth cohorts. We investigated within- and between-phenotype differences compared with binary latent class analysis models and ascertained associations of these phenotypes with asthma and lung function and with polymorphisms in asthma loci 17q12–21 and *CDHR3* (cadherin-related family member 3).

**Measurements and Main Results:**

Analysis among 7,719 participants with complete data identified five spell-based wheeze phenotypes with a high degree of certainty: never (54.1%), early-transient (ETW) (23.7%), late-onset (LOW) (6.9%), persistent (PEW) (8.3%), and a novel phenotype, intermittent wheeze (INT) (6.9%). FEV_1_/FVC was lower in PEW and INT compared with ETW and LOW and declined from age 8 years to adulthood in INT. 17q12–21 and *CDHR3* polymorphisms were associated with higher odds of PEW and INT, but not ETW or LOW. Latent class analysis- and spell-based phenotypes appeared similar, but within-phenotype individual trajectories and phenotype allocation differed substantially. The spell-based approach was much more robust in dealing with missing data, and the derived clusters were more stable and internally homogeneous.

**Conclusions:**

Modeling of spell variables identified a novel intermittent wheeze phenotype associated with lung function decline to early adulthood. Using multidimensional spell variables may better capture wheeze development and provide a more robust input for phenotype derivation.

**At a Glance Commentary**

**Scientific Knowledge on the Subject**

Longitudinal modeling of current wheezing identified similar phenotypes, but their characteristics often differ between studies.

**What This Study Adds to the Field**

Transformation of binary wheeze data into a set of multidimensional variables better captures the temporal characteristics of wheeze development and provides a more robust input for phenotype derivation. Modeling using multidimensional variables of wheezing spells identified a stable and consistent architecture of wheezing illness, including a novel intermittent phenotype associated with early lung function decline to early adulthood. Different wheezing phenotypes are underpinned by unique mechanisms and genetic associates.

Wheeze in most children remits by school age but in others may persist, with or without periods of remission. Over the past decades, a substantial effort has been devoted to understanding the heterogeneity of childhood wheezing illness, using both hypothesis-driven approaches, in which phenotypes are specified *a priori* based on clinical insights (1), and data-driven approaches, which incorporate a variety of multivariate statistical and machine learning methodologies (2). The latter have largely used latent class modeling, such as latent class analysis (LCA), in which repeated information of wheeze presence is used to uncover the temporal patterns over a specified time interval (3–15). These different symptom patterns may indicate distinct causes and biological mechanisms (16, 17), and their discovery may facilitate stratified treatment (18). However, to facilitate the identification of genetic associates and underlying mechanisms, phenotypes should be internally homogeneous and consistent between different populations and studies.

The number of phenotypes reported in previous analyses that used LCA varied by study, but four were identified in all cohorts (19): never or infrequent wheeze (NWZ), early-transient (ETW), late-onset (LOW), and persistent wheeze (PEW). Some analyses identified one or two further “intermediate” phenotypes (3, 4, 20), which mostly arose from transient or late-onset patterns (21). However, although phenotypes in different studies are usually designated with the same name, they often differ in temporal trajectories, distributions within a population, and associated risk factors (19, 22). These differences are in part a consequence of the sample size and the timing and frequency of data collection (21). Furthermore, the confidence with which individuals are assigned to a phenotype varies across phenotypes, and a substantial number of children in such analyses are classified imprecisely (e.g., individuals with identical wheeze patterns may be assigned to different phenotypes, or individual trajectories may not follow wheeze patterns suggested by the phenotype label [13, 21, 23]).

We propose that within-class heterogeneity and inaccurate allocation of individual children may, in part, be responsible for a lack of consistent associations of discovered phenotypes with risk factors (24) and may adversely impact the ability to identify phenotype-specific genetic associates and underlying mechanisms. We hypothesize that incorporating a more comprehensive description of wheeze may better describe wheeze trajectories and derive more within-phenotype homogeneity to facilitate a better understanding of their differing etiology. To address our hypothesis, we drew on research in other fields, specifically the “spells” approach pioneered in the social sciences research on poverty dynamics (25–28), to move from the point prevalence of current wheeze to a dynamic approach that takes into account the duration of wheezing spells, their temporal sequencing, and the extent of persistence and recurrence (further details can be found in the online supplement). To this end, we first developed a set of multidimensional variables to describe more comprehensively the temporal variation of wheeze and then applied a clustering approach based on the partition-around-medoids (PAM) algorithm (29) on these variables. We then investigated variation within and between phenotypes from binary (LCA) and indicator-based (PAM) models to ascertain whether we achieved increased within-phenotype homogeneity and investigated the associations of the derived clusters with early-life factors and asthma-related outcomes in adolescence. Finally, we tested the hypothesis that phenotypes defined using this approach have distinct genetic associates by investigating their associations with the known asthma loci (17q12–21 and *CDHR3*).

## Methods

### Study Design, Setting, and Participants

The Study Team for Early Life Asthma Research (STELAR) consortium (30) brings together five UK population-based birth cohorts: ALSPAC (Avon Longitudinal Study of Parents and Children) (31), Ashford (32), IOW (Isle of Wight) (33), and SEATON (Aberdeen) (34) cohorts, and the MAAS (Manchester Asthma and Allergy Study) (35). The cohorts are described in detail in the online supplement. All studies were approved by research ethics committees. Informed consent was obtained from parents, and study participants gave their assent or consent when applicable. Data were harmonized into the web-based knowledge management platform to enable joint analyses (30).

### Data Sources and Definition of Variables

Validated questionnaires were completed on multiple occasions from infancy to adolescence (23). The cohort-specific time points and sample sizes are shown in Table E1 in the online supplement. For the analyses of pooled data, we defined epochs based on the data availability at shared time points across cohorts: infancy (0.5–1 yr); early childhood (2–3 yr); preschool to early school (4–5 yr); middle childhood (8–10 yr); and adolescence (14–18 yr) (23). For each child, we derived six wheeze variables:

1. Age of the first episode.
2. Age of the last recorded episode.
3. Total number of separate records over the observation period.
4. Duration of the longest spell based on the number of consecutive records of wheeze.
5. Total number of separate wheeze spells.
6. Spell type: a categorical variable defined as 0 = no wheeze, 1 = single spell, and 2 = intermittent spells (defined as at least two nonconsecutive spells of wheeze of any length).

An illustrative example of the derivation of the variables is shown in Table E2.

We performed spirometry in adolescence in all cohorts and ALSPAC and MAAS on at least three follow-ups from school-age to early adulthood. We recorded FEV_1_ and FVC and expressed data as *z*-scores for each population.

Skin testing was performed in early to midschool-age in all cohorts and on six follow-ups in MAAS. The definitions of all variables can be found in the online supplement.

### Statistical Analysis

We analyzed pooled data from participating cohorts. Figure E1 provides an overview of the analytical steps. A detailed description is provided in the online supplement.

#### Wheeze phenotypes from infancy to adolescence from six derived variables

To derive longitudinal wheeze patterns captured by the multidimensional variables, we used the PAM (29) algorithm coupled with the Wishart distance for mixed data (36), initially among 7,719 participants with complete data on wheezing at all five time points. To investigate whether our findings were influenced by missing data, we adopted the framework of Basagaña and colleagues (37), which integrates multiple imputations (38) into cluster analysis, and applied it to data of 15,848 participants with at least two observations.

#### Comparison of wheeze phenotypes derived using binary LCA and spell PAM approaches

We first repeated analyses from our previous study, which used LCA to identify five wheeze phenotypes in the same 7,719 participants (never or infrequent, preschool remitting, midchildhood remitting, persistent, and late-onset) (23), and assigned participants to phenotypes according to the maximum posterior probability. We then compared the within-class homogeneity of both models. We checked the stability of cluster allocations using the adjusted Rand index (39) and plotted the magnitude of transitions of phenotype membership between models using alluvial plots.

#### Association of spell-based PAM phenotypes with early-life risk factors and clinical outcomes in adolescence

We used multinomial logistic regression to ascertain early-life associates of each PAM phenotype and examine their relationship with doctor-diagnosed asthma and asthma medication use in adolescence; results are reported as relative risk ratios with 95% confidence intervals (CIs). Associations with lung function (z-scores for FEV_1_, FVC, and FEV_1_/FVC adjusted for height, age, and sex) were investigated using linear regression. Models were adjusted for potential confounders, including maternal history of asthma, maternal smoking, and low birth weight.

#### Genetic associates of spell-based PAM phenotypes

We investigated the association of derived clusters with 17q12–21 SNPs (Table E3) and *CDHR3* SNP rs6967330 (40). We selected one representative 17q12–21 SNP per linkage disequilibrium block, leaving rs7216389, rs4795408, and rs3894194 in the final analysis. We tested the additive model using multinomial logistic regression.

## Results

### Characteristics of the Study Population

Of 7,719 children with complete data on wheezing, 50.4% were male. At the follow-up in adolescence, 12.9% had current asthma, and 11.4% reported using asthma medication. Demographic characteristics are shown in Table E4 and wheeze prevalence in Table E5. The prevalence of current wheeze decreased from 22.8% in infancy to 13.7% in adolescence.

### Wheeze Phenotypes Obtained Using Six Derived Variables and PAM Algorithm

**Figure 1.**
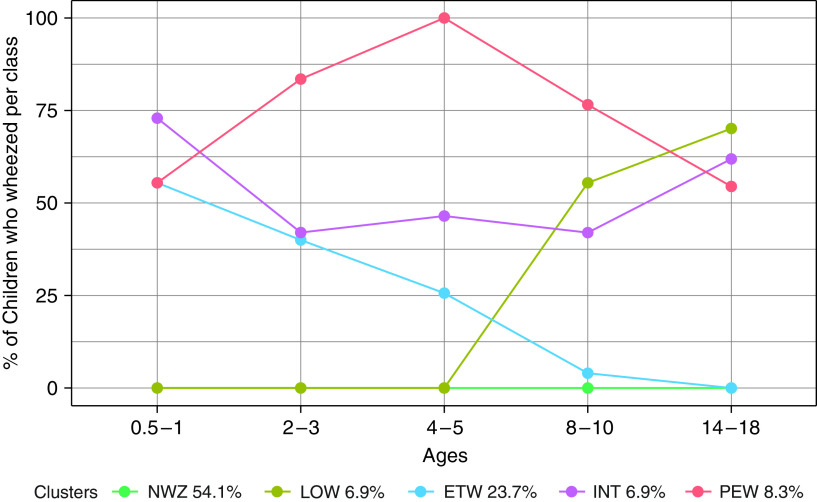
Trajectories of five wheeze classes were obtained with the partition-around-medoids algorithm: percentage of participants with reported wheezing in each time interval in the five cohorts. ETW = early transient; INT = intermittent; LOW = late onset; NWZ = never wheeze; PEW = persistent.

A five-cluster solution was selected as the optimal based on statistical fit (Figure E2). After inspection of trajectories for each cluster (Figure 1), the clusters (phenotypes) were characterized as *1*) NWZ (54.1%); *2*) ETW (23.7%); *3*) LOW (6.9%); *4*) PEW (8.3%); and *5*) INT wheeze (6.9%). The same five-class structure was evident when we modeled each cohort separately (Table E6), and the optimal solution was stable to changes in sample size (Table E7).

#### Impact of missing data on cluster derivation

Detailed analysis is shown in the online supplement. The optimal solution from the model using 15,848 individuals with at least two observations was very similar to that from 7,719 participants with complete data (Table E8). Children were assigned to clusters with a high degree of certainty (Table E9). There was a very high agreement between phenotype assignment of individual children when using complete or imputed data (adjusted Rand index = 0.94); only 195 of 7,719 (2.5%) children changed phenotype allocation (Figure E3).

### Comparison of Wheezing Phenotypes Derived Using Binary LCA and Spell PAM Approaches

**Figure 2.**
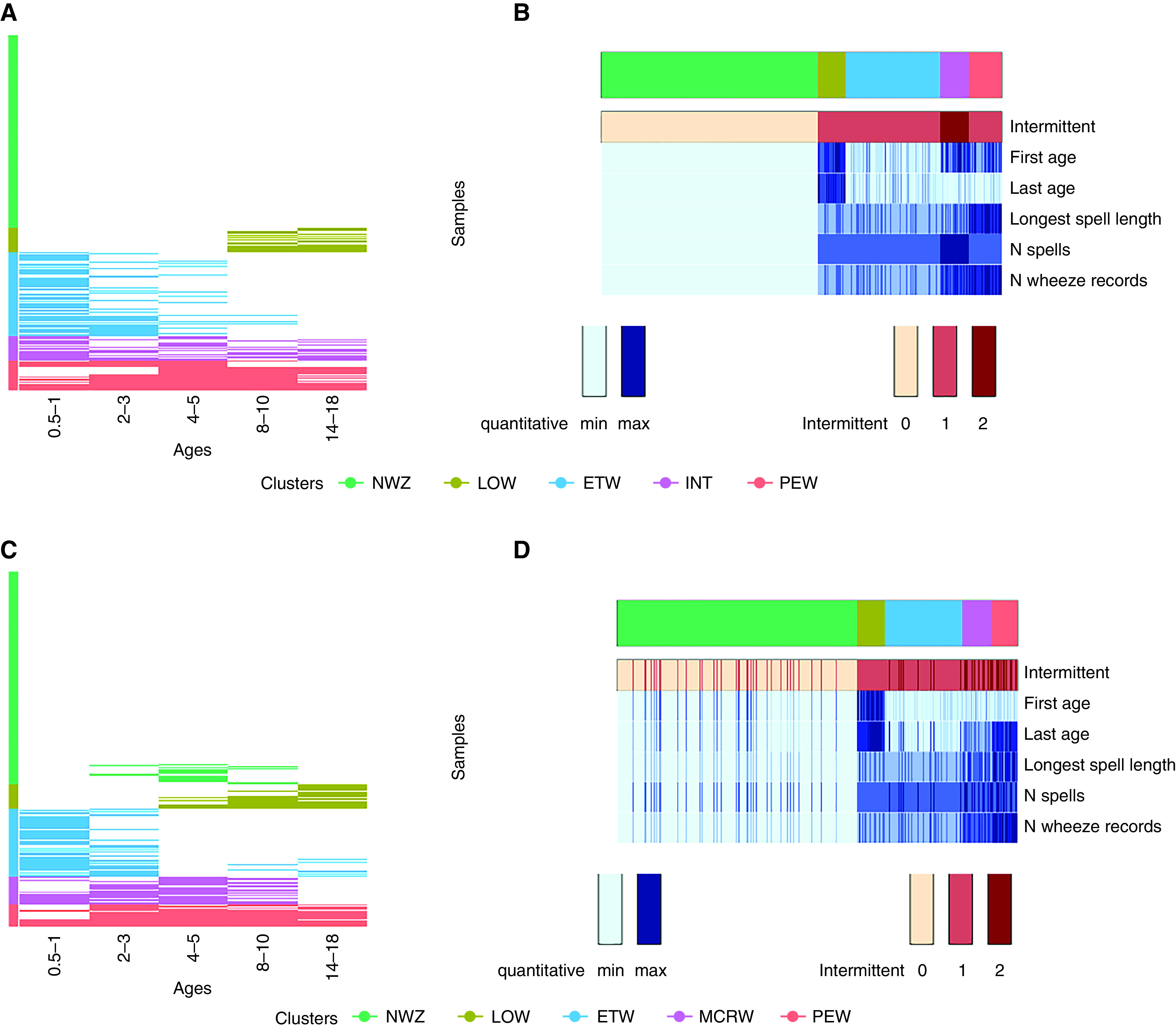
(*A*–*D*) Comparison of internal homogeneity of wheezing phenotypes derived using the spells partition-around-medoids (*A* and *B*) and binary latent class analysis approaches (*C* and *D*) among 7,719 subjects with complete data on wheezing from infancy to adolescence. (*B* and *D*) Plots are multidimensional heatmaps that show the density of the distribution of each of the six derived variables, each of which is represented as a row. The scale of the variables (quantitative and categorical) is shown at the bottom of the plot. The segments in the top bar represent each cluster and its relative size. The distribution of each indicator within each cluster is shown vertically. In *B*, for example, intermittent spells (as represented by category two for the intermittent variable) are only present in the pink INT cluster; in *D*, the latent class analysis model, intermittent wheeze is present in all classes. ETW = early transient; INT = intermittent; LOW = late onset; MCRW = midchildhood remitting wheeze; NWZ = never wheeze; PEW = persistent. For intermittent, 0 = no wheeze, 1 = single spell, and 2 = intermittent spells.

Figure E4 shows latent classes (phenotypes) identified by LCA. Phenotypes derived using the two methods among the same 7,719 participants appeared very similar, and four appeared identical (NWZ, ETW, PEW, and LOW) (Figures 1 and E4). However, the within-phenotype structure differed substantially (Figure 2). For example, in PAM-NWZ, no participants reported wheezing at any time point (Figure 2A), whereas in LCA-NWZ, 10% reported occasional wheezing (Figure 2C). In PAM-ETW, no participants reported wheezing after age 10 years, and nobody in PAM-LOW wheezed before age 8; in contrast, in the LCA-ETW class, 8% reported wheeze up to age 18 years, and wheeze before age 10 was present among 42% in LCA-LOW.

Figures 2B and 2D and Table E10 show the distribution of wheeze variables between phenotypes from the two approaches. In PAM-LOW, the earliest observed age of wheeze onset was 7 years later than in LCA-LOW. PAM-PEW only contained children with a long single spell of wheeze, whereas subjects in the LCA-PEW also had intermittent spells.

We further investigated the differences between individual allocations to PAM and LCA phenotypes for all 32 possible wheeze sequences across the five time points (Table E11). We did not observe any inconsistencies across cohorts in the PAM model (i.e., the same sequences were always assigned to the same cluster). In contrast, children with identical sequences were assigned by LCA to different phenotypes (e.g., “0–1-0–1-0” was assigned to three different LCA phenotypes, whereas PAM spell-based analysis always assigned this sequence to the INT phenotype).

Figure E5 shows differences in individual assignment to PAM and LCA phenotypes. One-quarter of subjects transitioned to a different phenotype. Higher stability was observed for ETW and LOW (>70%) but was relatively poor in the PEW (60%). Children in the PAM-INT cluster transitioned from all LCA phenotypes.

Finally, we applied the PAM algorithm to the binary current wheeze variable (yes or no) to investigate whether the algorithm or the transformation to spell-based variables gave rise to homogeneous phenotypes. A five-cluster solution was optimal; however, the clusters resembled LCA phenotypes (with no INT wheeze) and were structurally internally much more heterogeneous than phenotypes obtained using the derived variables (Figure E6). Therefore, it is likely that the derived variables were, primarily, the precursor for deriving more homogeneous phenotypes.

### Association of Spell-based Phenotypes with Early-Life Risk Factors and Asthma-related Outcomes

#### Family history, early-life factors, and environmental exposures

Univariable analyses are shown in Table E12. Table E13 shows the results of multivariable logistic regression models. Males had a higher risk of developing PEW, ETW, and INT, but not LOW. Maternal asthma and parental smoking were associated with all four clusters. Low birth weight was associated with ETW, INT, and PEW (with the strongest association with PEW) but not with LOW.

#### Asthma

**Figure 3.**
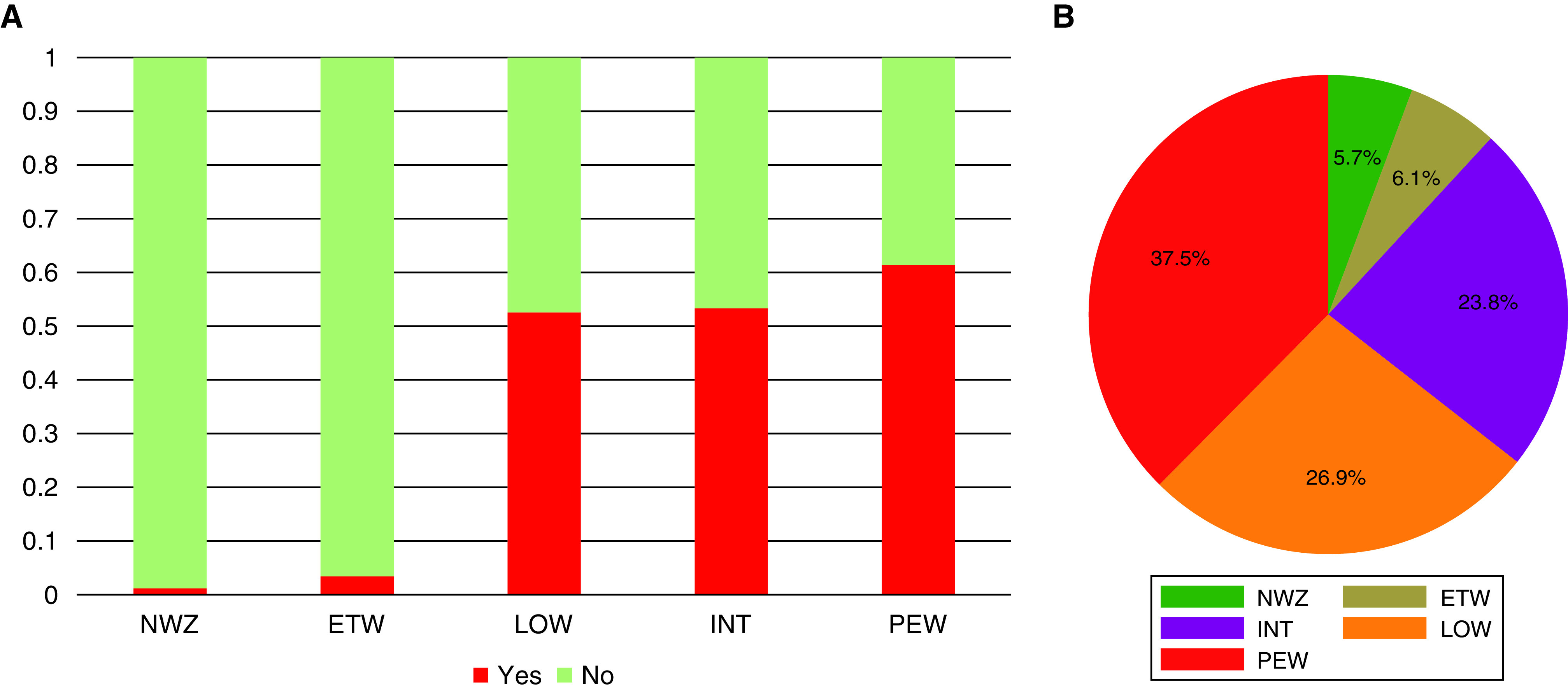
(*A* and *B*) The proportion of study participants with asthma diagnosis in adolescence in each partition-around-medoids wheeze phenotype (*A*) and the proportion of subjects with asthma diagnosis in adolescence belonging to each partition-around-medoids phenotype (*B*). ETW = early transient; INT = intermittent; LOW = late onset; NWZ = never wheeze; PEW = persistent.

Compared with NWZ, all four wheeze clusters were associated with a higher risk of asthma diagnosis and medication use in adolescence (Table 1). The associations were strongest for PEW and weakest for ETW (e.g., the risk of using asthma medication was approximately 14-fold higher for PEW than ETW). Variability in the proportion of asthmatics by spell-based phenotype and the proportion of subjects with asthma diagnosis in adolescence in each phenotype is shown in Figure 3; of note, 5.7% of children with asthma diagnosis in adolescence never reported wheezing.

**Table 1.** Associations of Wheezing Phenotypes with Asthma-related Outcomes in Adolescence

	Associations with Asthma in Adolescence*				Associations with Lung Function in Adolescence*		
	Current^†^ Asthma	Asthma Ever	Current^†^ Asthma Medication	Asthma Medication Ever	z-Scores for FEV_1_^‡^	z-Scores for FVC^‡^	z-Scores for FEV_1_/FVC^‡^
Never wheeze	Reference	Reference	Reference	Reference	Reference	Reference	Reference
Early transient	2.44 (1.84 to 3.24)	4.00 (3.45 to 4.63)	1.96 (1.48 to 2.58)	3.31 (2.92 to 3.75)	–0.103 (–0.19 to –0.02)	–0.014 (–0.10 to 0.07)	–0.151 (–0.24 to –0.07)
*P* value	**<0.0001**	**<0.0001**	**<0.0001**	**<0.0001**	**0.021**	0.748	**<0.0001**
Intermittent	27.06 (20.44 to 35.84)	22.77 (18.23 to 28.44)	17.34 (13.17 to 22.84)	18.47 (15.21 to 22.43)	–0.168 (–0.29 to –0.05)	0.054 (–0.06 to 0.17)	–0.379 (–0.49 to –0.27)
*P* value	**<0.0001**	**<0.0001**	**<0.0001**	**<0.0001**	**0.005**	0.37	**<0.0001**
Persistent	37.72 (29.13 to 48.85)	48.34 (38.47 to 60.74)	26.78 (20.90 to 34.32)	38.97 (32.15 to 47.24)	–0.326 (–0.45 to –0.20)	0.079 (–0.05 to 0.21)	–0.707 (–0.83 to –0.59)
*P* value	**<0.0001**	**<0.0001**	**<0.0001**	**<0.0001**	**<0.0001**	0.221	**<0.0001**
Late onset	35.44 (27.30 to 46.00)	17.8 (14.58 to 21.73)	16.78 (12.96 to 21.72)	22.32 (18.26 to 27.27)	–0.003 (–0.13 to 0.13)	0.159 (0.03 to 0.29)	–0.302 (–0.43 to –0.18)
*P* value	**<0.0001**	**<0.0001**	**<0.0001**	**<0.0001**	0.959	**0.015**	**<0.0001**

Results are from a multinomial logistic regression using children with at least two observations on wheeze (reference class: never wheeze) using weighted membership probabilities. Weights derived from probabilities of class membership across 10 imputation samples from the partition-around-medoids model. Results are reported as adjusted odds ratios with 95% confidence intervals. Bold figures indicate statistically significant differences at *P* < 0.05.

* Models adjusted for maternal history of asthma (recruitment), maternal smoking (recruitment), and low birth weight.

^†^ Available at the latest follow-up (18 years in Isle of Wight, 16 years in the Manchester Asthma and Allergy Study, 15 years in Aberdeen, 15 years in Ashford, and 15 years in the Avon Longitudinal Study of Parents and Children).

^‡^ Sex-, age-, and height-adjusted SD units.

#### Allergic sensitization

**Figure 4.**
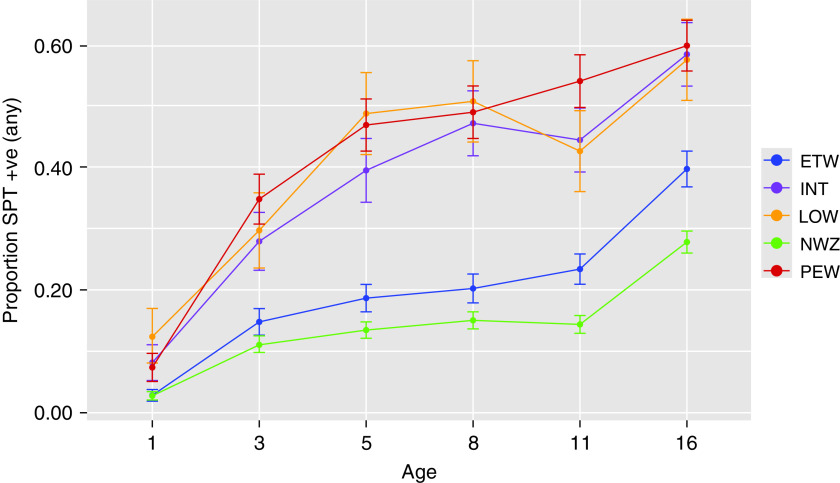
The proportion of children with allergic sensitization in each partition-around-medoids wheeze cluster (Manchester Asthma and Allergy Study). ETW = early transient; INT = intermittent; LOW = late onset; NWZ = never wheeze; PEW = persistent; SPT +ve = sensitized to at least one allergen based on a positive skin prick test.

All phenotypes were associated with sensitization in early school-age (Table E13), with the magnitude of risk being higher for PEW and LOW. Trajectories of sensitization from infancy to adolescence in MAAS were almost identical in PEW, INT, and LOW and differed from those in NWZ and ETW (Figure 4) (i.e., highly concordant longitudinal sensitization patterns were associated with different wheeze phenotypes). In general, wheeze preceded sensitization in PEW and INT clusters, whereas sensitization preceded wheeze in LOW.

#### Lung function

**Figure 5.**
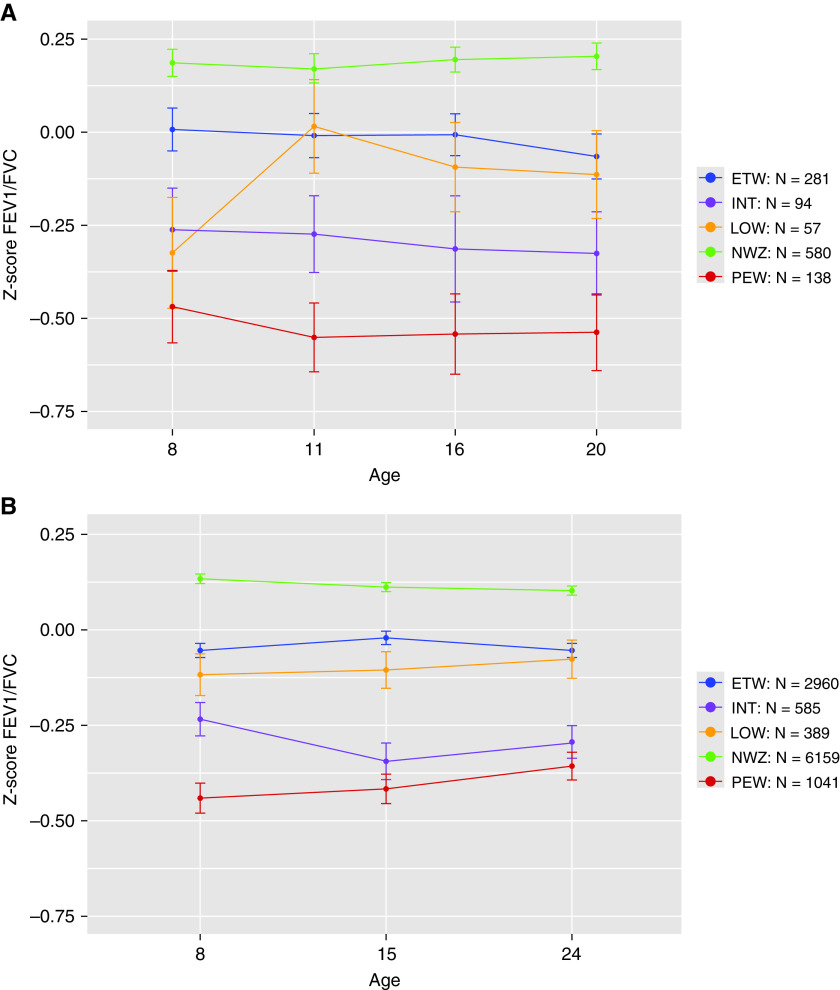
(*A* and *B*) Lung function trajectories from early school-age to early adulthood in Manchester Asthma and Allergy Study (*A*) and Avon Longitudinal Study of Parents and Children (*B*). ETW = early transient; INT = intermittent; LOW = late onset; NWZ = never wheeze; PEW = persistent.

FEV_1_/FVC in adolescence was lower in all four wheeze phenotypes compared with children who never wheezed, with those in PEW having the lowest lung function, markedly lower compared with NWZ (*z*-score, −0.71; 95% CI [−0.83 to −0.59]; *P* < 0.0001) (Table 1). FVC was similar across clusters. Longitudinal lung function was available in 6,729, 4,567, and 3,749 participants at ages 8, 15, and 24 years, respectively, in ALSPAC, and 790, 801, 630, and 504 participants at ages 8, 11, 16, and 20 years, respectively, in MAAS. FEV_1_/FVC was significantly lower in all wheeze phenotypes compared with NWZ throughout the follow-up (Figure 5) and was consistently lower in PEW and INT compared with ETW and LOW (Table E14). FEV_1_/FVC declined from age 8 years to early adulthood in INT, but not other phenotypes.

### Association between Spell-based Phenotypes and Genetic Variants in 17q12–21 and *CDHR3*

**Figure 6.**
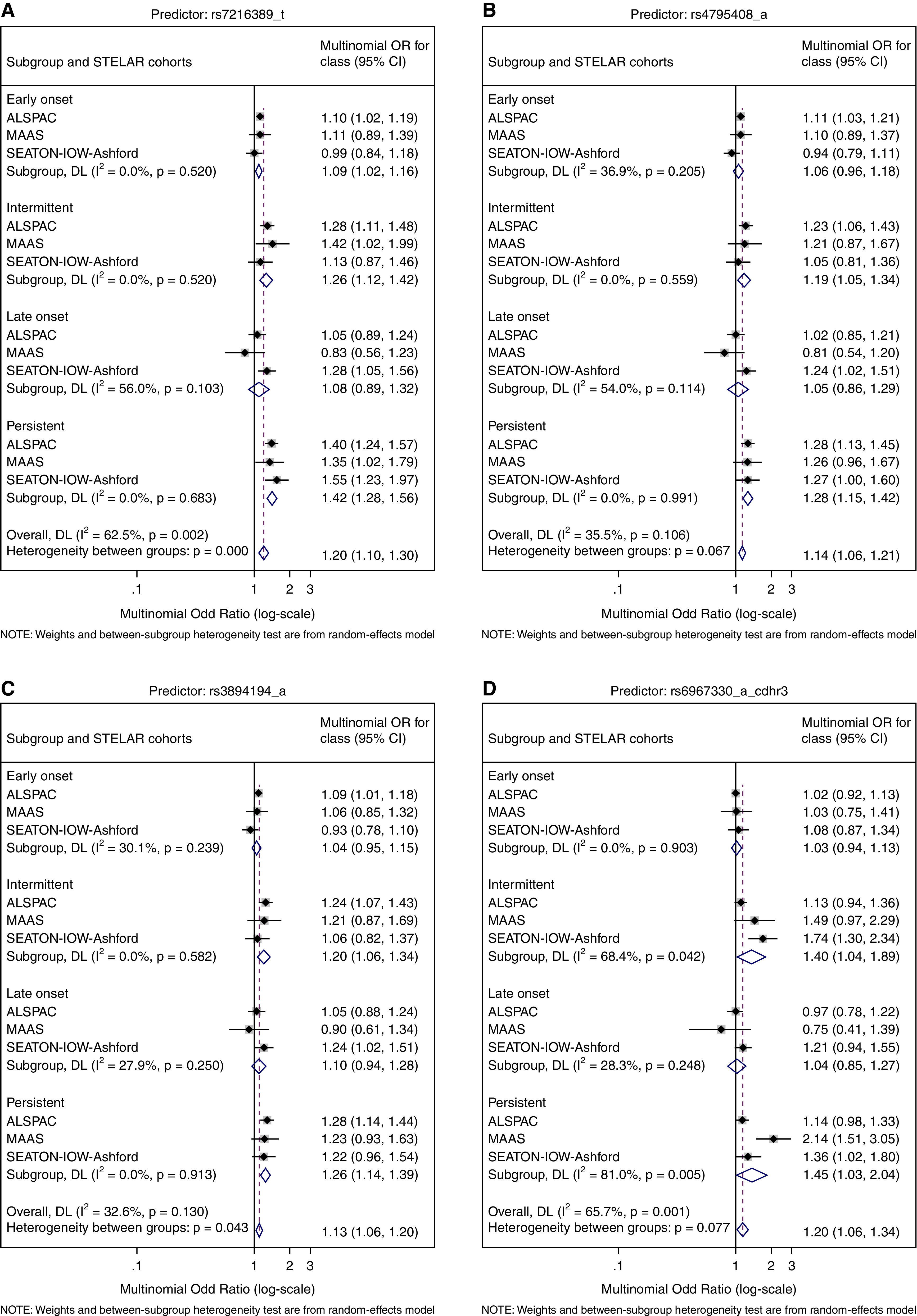
(*A*–*D*) Forest plots of associations of 17q12–21 SNPs (*A–C*) and *CDHR3* (cadherin-related family member 3) (*D*) with partition-around-medoids wheeze clusters. ALSPAC = Avon Longitudinal Study of Parents and Children; CI = confidence interval; DL = DerSimonian–Laird method for estimating between-study variability; IOW = Isle of Wight; MAAS = Manchester Asthma and Allergy Study; OR = odds ratio; SEATON = Aberdeen; STELAR = Study Team for Early Life Asthma Research.

Subjects of White European ancestry (9,655) had genotyping data and were included in the meta-analysis of genetic associations. Figure 6 shows forest plots of the associations for representative SNPs. Subgroup-level *P* values are presented in Table E15. We found strong evidence of an association between all 17q12–21 SNPs and PEW. INT was also associated with 17q12–21 SNPs. However, we found little evidence of an association between 17q12–21 SNPs and ETW and LOW.

We found strong evidence of an association between *CDHR3* SNP rs6967330 and PEW (odds ratio, 1.45; 95% CI [1.03–2.04]) and INT (odds ratio, 1.40; 95% CI [1.04–1.89]), but there was no association with ETW and LOW clusters.

## Discussion

We applied a framework that focused on wheezing spells to describe the temporal patterns of wheeze from infancy to adolescence. Our results suggest that this approach better captures wheeze development than the presence or absence of wheezing alone and provides a more robust input for data-driven phenotype derivation. It is much more robust in dealing with missing data, and the derived clusters are stable and internally homogeneous. Our spell-based analysis applied to data from five population-based birth cohorts identified a novel wheezing phenotype, intermittent wheeze, to which ∼7% of participants were assigned. FEV_1_/FVC trajectory from school-age to physiological peak in early adulthood showed consistently diminished lung function in all four wheeze phenotypes determined using the spell-based approach compared with never wheezers, and in persistent and intermittent compared with transient-early and late-onset wheezing. Lung function declined from age 8 years to early adulthood in intermittent, but not other phenotypes. Finally, associations with 17q12–21 and *CDHR3* SNPs differed across wheezing phenotypes, and carriers of risk variants had significantly increased risk for persistent and intermittent, but not of transient or late-onset wheeze.

Wheezing phenotypes developed using spells appeared more clinically intuitive than those derived based on wheeze presence or absence. For example, no subjects in spell-based ETW reported wheezing after age 10 years, and nobody in LOW wheezed before age 10 years; in contrast, in the LCA-ETW, some children reported wheeze to age 18 years, and early-life wheeze was reported in some individuals assigned to LCA-LOW. In spell-based LOW, the earliest observed age of wheeze onset was 7 years later than in LCA-LOW.

Within-class heterogeneity may dilute associations with biomarkers, genetic variants, and environmental factors. Therefore, for such analyses, phenotypes derived using data-driven methods should be homogenous, and individual patterns of symptoms within each phenotype should be distinct from individuals in other subgroups. Our previous LCA showed that a substantial number of children are classified imprecisely using binary information on wheeze, particularly when an individual’s posterior probability of assignment is less than 0.80 (21). Similarly, a recent US study that derived wheeze phenotypes using LCA found that one-third of subjects had a posterior probability of less than 0.80 (13). Our current analysis demonstrates that when using the binary representation of wheeze, some wheeze patterns are not assigned to phenotypes with high precision, and consequently, individuals with the same longitudinal wheezing patterns can be assigned to different phenotypes. The intermittent patterns contributed to substantial within-class heterogeneity when using binary data in both LCA and PAM models. Once the spell approach isolated these intermittent patterns, ETW, LOW, and PEW were more internally homogeneous, and a novel INT cluster emerged.

Our previous analysis in the same study population showed that data imputation has a major impact on the assignment of individual participants to different phenotypes in LCA (e.g., ∼40% of children switched from early-onset middle-childhood remitting to PEW from the model with complete data to that with imputed data [23]). In contrast, in the current study, there was a remarkably high agreement between the assignment of individuals into clusters when using complete or imputed data, and only 2.5% of children changed phenotype. This is of key importance for longitudinal studies in which data missingness is inevitable and for genetic analyses in which a large sample size is essential.

The important question as to whether different longitudinal wheezing phenotypes are underpinned by unique pathophysiological mechanisms has been asked by Koppelman and Kersten (41) in an editorial following the recent finding from the CREW consortium, which investigated the association of 17q12–21 SNPs with LCA-derived phenotypes (13). In this study, contrary to the hypothesis of differential genetic associations of different wheeze phenotypes, associations between multiple 17q12–21 SNPs were similar for all LCA phenotypes, suggesting that all wheezing phenotypes have shared genetic origin in relation to this locus (13). In contrast, we found a clear differential association of genetic markers between phenotypes derived using spell-based variables. We found no association of the SNPs in this locus with transient and late-onset wheezing, and our results do not support the notion that the 17q locus should be considered a “wheezing locus.”

Both 17q21 locus and *CDHR3* are linked to differential susceptibility to infection by rhinoviruses (42, 43), and our data suggest that such susceptibility is common and important for early-onset nontransient phenotypes (both persistent and intermittent). However, most children who wheeze in early life stop wheezing by school-age (∼2/3 in our dataset, all of whom clustered to spell-based ETW), and known genetic markers of susceptibility to rhinoviruses were not apparent in this group. This is consistent with recent data showing that even among children with severe recurrent preschool wheeze, ∼50% had no evidence of either inflammation or infection in their lower airways (44). It is possible that diminished lung function in early childhood (as suggested by the seminal study from the Tucson cohort [45] and indirectly confirmed in one of our cohorts [46, 47]) is associated with poor growth in early childhood (48) or specific genetic susceptibility (49, 50) and is a principal cause of early-onset transient wheezing, whereas susceptibility to viruses may contribute to persistence and exacerbations. We cannot exclude that the immune response to other viruses (such as respiratory syncytial virus) may also be important in ETW (51). Our data also suggest that LOW (which in the current analysis started after age 10 yr) in most children may not be associated with susceptibility to viruses but is predominantly an allergic airway disease, as suggested by the analysis of the pattern of *in vitro* immune responses to viruses (52). In these individuals, allergen exposure may be the principal contributor to severity and exacerbations (53). However, it is important to emphasize that all wheeze phenotypes were associated with diminished lung function in adolescence and early adulthood, with the greatest impairment in PEW and INT. This is a precursor of chronic obstructive pulmonary disease (54–56), early all-cause mortality (57), and early-onset cardiovascular, respiratory, and metabolic comorbidities (58).

We found that 5.7% of children with asthma diagnoses in adolescence belonged to the NWZ group (and a similar proportion to the ETW group). This emphasizes the heterogeneity of doctor-diagnosed asthma at the population level and the fact that children with other respiratory symptoms such as cough (even in the absence of wheezing) are diagnosed as being asthmatic.

One limitation of our study is that the population is not ethnically diverse. In addition, early-life pulmonary or airway function tests were not performed, which limits the inference to the potential role of premorbid lung function. We also acknowledge that our study was not able to investigate the relationship between wheeze treatment, disease severity, and patterns of wheeze spells. With respect to genetic analyses, further investigations are needed at a genomewide level to help distinguish mechanisms of early-life wheeze and subsequent asthma.

In conclusion, our data are consistent with the notion that in addition to shared pathophysiology, distinct wheezing phenotypes are underpinned by unique mechanisms and genetic associates. Modeling using multidimensional variables of wheezing spells identified a stable and consistent architecture of wheezing illness, including a novel intermittent phenotype associated with early lung function decline to early adulthood. We suggest that the transformation of binary data into a set of multidimensional variables may better capture the temporal characteristics of wheeze development and may provide a more robust input for phenotype derivation.
